# Commentary: The Spinal Cord Has an Intrinsic System for the Control of pH

**DOI:** 10.3389/fphys.2016.00513

**Published:** 2016-11-07

**Authors:** Joseph M. Santin, Tobias Wang, Saihari S. Dukkipati, Lynn K. Hartzler

**Affiliations:** ^1^Department of Biological Sciences, Wright State UniversityDayton, OH, USA; ^2^Department of Bioscience, Zoophysiology, Aarhus UniversityAarhus, Denmark; ^3^Department of Neuroscience, Cell Biology, and Physiology, Wright State UniversityDayton, OH, USA

**Keywords:** pH, spinal cord, temperature, alpha-stat, CSF-c neuron

Most physiological processes are sensitive to pH (i.e., hydrogen ion activity) and regulation of pH within tissues and body fluids represents a fundamental homeostatic process in living organisms. At the organismal level, acid-base balance is achieved by fast respiratory modulations of the partial pressure of CO_2_ in combination with slower transepithelial ion movements to accommodate excretion and retention of bicarbonate and protons in response to acid-base disturbances. At the cellular and subcellular levels, acid-base balance is achieved by ion exchange.

Recently in Current Biology, Jalalvand et al. (2016) described a novel pH sensing system in the spinal cord of lampreys with an ability to inhibit locomotor activity. This system involves inhibitory spinal neurons in intimate contact with the cerebrospinal fluid, termed CSF-c neurons that increase firing frequencies whenever pH deviates in either direction from 7.4. Due to their inhibitory nature, activation of the CSF-c neurons decreases locomotor activity when pH strays above and below 7.4. This presents a unique and unusual mechanism whereby extracellular pH exerts direct regulation of locomotor activity. The authors conclude that this sensory system represents a “novel innate homeostatic mechanism, designed to sense any deviation from physiological pH and to respond by causing a depression of the motor activity” (Jalalvand et al., 2016). In the following, we argue that the pH sensory system uncovered by Jalalvand et al. does not support this conclusion because the pH of 7.4—interpreted as the control condition—is incorrect for ectothermic animals, such as lampreys when studied at low body temperature (Wang and Jackson, 2016).

The authors erroneously assume that a superfusate pH of 7.4 mimics the normal pH of body fluids in ectotherms at 8–10°C, the temperature where the *in vitro* spinal cord was studied. While mammals regulate arterial blood pH (pH_a_) at 7.4 at their normal body temperature of 37°C, Jalalvand et al. (2016) ignore that both pH_a_ and CSF pH (pH_CSF_) increase by ~0.015 unit per °C when body temperature decreases in ectothermic vertebrates (Burton, 2002). This so-called alphastat regulation serves to maintain protein ionization (Reeves, 1977), and explains why the normal and regulated pH_a_ of the closely related sea lamprey, *Petromyzon marinus*, is ~8.1 at 8–10°C (Tufts et al., 1992). As a consequence, the control value of 7.4 used by Jalalvand et al. is highly acidic, even when compared with maximal acidosis (pH_a_ 7.7) upon intense and exhaustive exercise (Tufts et al., 1992). Ectothermic vertebrates, including aquatic species, have pH_CSF_ values ~0.1–0.2 pH units lower than pH_a_ at low temperatures (Hitzig, 1982; Wood et al., 1990). Assuming a pH_a_ value of ~8.1, pH_CSF_ should be ~7.9 in resting lampreys.

The much higher *in vivo* pH than that used in the *in vitro* experiments completely alters the interpretation of the “U-shaped curve” with minimum firing frequencies of CSF-c neurons at 7.4 (Jalalvand et al., 2016). Rather than demonstrating minimum CSF-c neuron activity at resting *in vivo* pH values, the true resting values are, in fact, on the right-hand side of ascending part of the “U” (see Figure 4F of Jalalvand et al., 2016). CSF-c neurons, therefore, offer no protective or homeostatic influence by minimizing locomotion when pH deviates in either direction from its resting value because a realistic acidosis of fluid in contact with these neurons would **decrease** inhibitory tone of the locomotor network, and hence **increase** locomotion.

We propose that CSF in the lamprey would be less acidic *in vivo* compared to the *in vitro* conditions used by Jalalvand et al. and this also applies to the pH of the interstitial fluid (pH_ins_) that interfaces with neuronal membranes. Chesler (1986) showed the lamprey brain *in vitro* has a pH_ins_ of 7.3–7.4 when bathed at 7.8 at 23°C, a control pH that is slightly acidic, but more appropriate than 7.4 for aquatic ectotherms (Wood et al., 1990). Thus, pH_ins_ values near ~7.5 would be expected if the control bathing solution had a correct pH of ~7.9. A large interstitial acidosis relative to the superfusate is anticipated *in vitro* since these preparations lack blood flow to remove metabolically produced CO_2_. Accordingly, pH_ins_ *in vivo* typically rest only ~0.1–0.2 pH unit below pH_a_ (Kraig et al., 1983). If we interpret the bath-interstitial pH difference observed *in vitro* as being, at least in part, physiological, this translates to pH_ins_ values of ~7.0, as measured by Chesler (1986), in contact with CSF-c neurons in the experiments by Jalalvand et al. Minimum frequencies of CSF-c neurons, therefore, center on a highly acidic pH_ins_ instead of a more alkaline value expected for lamprey. Since minimum frequencies of CSF-c neurons occur at acidic values, this sensor might, instead, operate normally along an alkaline-activated and acid-inhibited slope that increases locomotor burst frequency during physiological acidification.

Jalalvand et al. did not state whether CSF-c neurons sample CSF or interstitial pH, making it unclear which compartment might have its pH controlled through pH sensing in CSF-c neurons and subsequent alterations in locomotion. Nor did they report what *in vivo* pH set points should be for any of these compartments. Disturbances in pH_ins_ arise during neuronal activity (Chesler, 2003), implying that CSF-c neurons could function on a slope within the physiological range. In contrast, it is less obvious what scenarios may change pH_CSF_ to alter firing rates of CSF-c neurons because metabolic acid-base disturbances are unlikely to alter pH_CSF_, at least in the short term, because the CSF is separated by the blood-brain barrier.

As articulated by Reeves (1977), “The very large number of investigations that uncritically used pH 7.4 for Ringer's solutions at any temperature in experiments on frog and other ectothermic tissue attests to how cherished misinformation can be even in the scientific community.” Until experiments are performed to clarify which compartments' pH may be determined by pH sensing in CSF-c neurons and under what conditions this sensor operates, it remains disputable that CSF-c neurons provide an “innate homeostatic mechanism” by inhibiting locomotion during deviations from physiological pH.

## Author contributions

JS, TW, SD, and LH interpreted results; JS wrote the manuscript; JS, TW, SD, and LH edited, revised, and approved final manuscript.

## Funding

We would like to thank the Wright State University Biomedical Sciences Ph.D. Program (JS), the Danish Natural Sciences Research Council (FNU) (TW), the National Institutes of Health- NS091836 to Sherif Elbasiouny (SD), and the National Science Foundation IOS-1257338 (LH) for funding.

### Conflict of interest statement

The authors declare that the research was conducted in the absence of any commercial or financial relationships that could be construed as a potential conflict of interest.
